# Cardiovascular outcomes in patients treated with sodium-glucose transport protein 2 inhibitors, a network meta-analysis of randomized trials

**DOI:** 10.3389/fcvm.2022.1041200

**Published:** 2022-12-05

**Authors:** Dániel Tornyos, Maximilian Meuer, Réka Lukács, Oumaima El Alaoui El Abdallaoui, Péter Kupó, Réka Faludi, András Komócsi

**Affiliations:** Heart Institute, Medical School, University of Pécs, Pécs, Hungary

**Keywords:** sodium glucose co-transport-2 (SGLT2) inhibitors, cardiovascular event, network meta-analysis, mortality, major adverse cardiac events (MACE), heart failure

## Abstract

**Background:**

Gliflozins altering the sodium-glucose transport protein 2 (SGLT2) in the nephron, represent alone or in combination a promising treatment option for patients with type II diabetes mellitus. In addition to glucose control, these drugs provide benefits including reduced risk of long-term cardiovascular (CV) and renal complications. Several trials evaluated gliflozins in patients with various degrees of cardiac dysfunction with heterogeneous results.

**Objectives:**

We aimed to perform a comprehensive analysis of the effect of gliflozins on CV outcomes.

**Methods:**

Systematic searches of electronic databases were conducted until September 2022. Multiple treatment network meta-analysis was performed in R. Random-effects model was used to combine risk estimates across trials calculating risk ratio (RR) with 95% confidence intervals as summary statistics. The primary endpoint of interest was the rate of heart failure-related hospitalization (HHF) and the composite of HHF with CV mortality (HHF + CVD). Secondary outcomes included major adverse cardiac events (MACE), CV- and overall mortality, myocardial infarction (MI), and stroke.

**Results:**

Twenty-nine studies randomizing 88,418 patients were identified. Gliflozins reduced the risk of HHF (RR: 0.72 [0.69; 0.76]) and HHF + CVD (RR: 0.78 [0.75; 0.82]). The risk of MACE and its component also improved significantly except for stroke. The network analyses did not explore major differences among the individual substances. The only exception was sotagliflozin which appeared to be more effective regarding HHF + CVD, stroke, and MI compared to ertugliflozin, in HHF + CVD and stroke compared to dapagliflozin, and in stroke endpoint compared to empagliflozin.

**Conclusion:**

Our meta-analysis supports a group effect of gliflozins beneficial in a wide spectrum of patients with a risk of heart failure (HF) development. In addition to the improvement of HF-related outcomes, the risk of major adverse events is also reduced with SGLT2 inhibition.

**Systematic review registration:**

[www.ClinicalTrials.gov], identifier [CRD42022358078].

## Introduction

Gliflozins inhibiting the sodium-glucose transport protein 2 (SGLT2) in the nephron, represent alone or in combination a promising treatment option for patients with type II diabetes mellitus (T2DM). Importantly, in addition to the improvement of blood glucose control, some of these drugs have been shown to provide an important reduction of long-term complications including cardiovascular (CV) and renal benefits. Moreover, several trials evaluated gliflozins in patients with various degrees of cardiac dysfunction with heterogeneous results. Among these, in 4 large-scale phase 3 trials, empagliflozin, and dapagliflozin resulted in a significant reduction in heart failure-related hospitalization (HHF) and mortality in patients with reduced and also with preserved ejection fraction (EF) (1–4). It is also of note that the gain of heart failure (HF) patients in these trials were independent of their diabetes status (5).

A combined analysis of 3 placebo-controlled studies found that empagliflozin, canagliflozin, and dapagliflozin reduced the rate of renal and CV outcomes including major adverse cardiac events (MACE) among patients with T2DM (6). This latter effect was neutral among those with only multiple risk factors and mostly present in the subgroup of patients with established CV disease (6).

However, from the data of these trials, it is hard to abstract the benefits and risk profile of a specific agent. To identify the potentially disparate effect of different SGLT2 inhibitors we performed a systematic review with multiple treatment network meta-analysis (NMA). The goal of this analysis is to review the latest evidence regarding the CV benefit of SGLT2 inhibitors.

## Methods

The protocol of this systematic review was registered in the PROSPERO (International Prospective Register of Systematic Reviews) database. The data preparation and analyses were performed according to the standards outlined in the PRISMA Extension Statement for Reporting of Systematic Reviews Incorporating Network Meta-analyses of Health Care Interventions (7). To identify clinical trials collecting CV endpoints in patients with gliflozin treatment, electronic searches were performed in the PubMed, Scopus, and the Cochrane Library database. Search queries were done without language restrictions and references to related articles between the inception of the database and September 1, 2022 were collected. The keywords included “Randomized Clinical Trial,” “SGLT-2 inhibitor,” “canagliflozin,” “dapagliflozin,” “empagliflozin,” “ertugliflozin,” “ipragliflozin,” “luseogliflozin,” “remogliflozin,” “rongliflozin,” “sergliflozin,” “sotagliflozin,” and “tofogliflozin.”

Titles and abstracts were scanned as well as the full-text screening was performed by 3 investigators against eligibility criteria as outlined in the PICO framework as “in patients at substantial risk for acute CV events including cases with a history of diabetes, coronary, or peripheral artery disease, or HF (P), whether an intervention with gliflozins (I) compared to placebo or different antidiabetic drug or combination (C) has a favorable effect on prognostically relevant outcomes defined as MACE and HF (O).” Studies were included if the following criteria were fulfilled: (a) randomized controlled clinical trials (RCT), (b) assessing the clinical efficacy and/or safety of gliflozin treatment (c) reported CV endpoints; CV mortality (CVD), myocardial infarction (MI), stroke, or HHF events from a minimum follow-up duration of 30 days. We excluded studies if any of the following criteria applied: (a) non-randomized or randomized studies with crossover design, (b) single-arm of dose-finding phase 2 studies, (c) if the outcomes of interest were not reported or were impossible to extract or calculate from the published results, or (d) duplicate publications. Data from subgroup analyses were only included if these were not readily available in the original publication of the complete trial. From publications of studies with multiple follow-ups, we selected those that best reflect the clinical result of a 12–24-month treatment period.

The selected publications including their supplementary materials were subject to data extraction using prestructured forms. Literature search and data extraction were performed independently by 3 authors with discrepancies resolved in consensus (DT, MM, AK).

We predefined the HHF and the composite of HHF with CVD (HHF + CVD) as primary efficacy outcomes of our analysis. Our secondary outcome was overall mortality. The occurrence of MACE was defined by the composite of CVD, MI, and stroke, and these individual components were also collected as secondary outcome measures. Internal definitions of the included trials were used for definitions of endpoint events. The endpoint data reported from the intention-to-treat analyses were extracted.

We used the Cochrane Collaboration tool for assessing the methodological quality of RCTs. Considering that the trials used different arms for comparing outcomes of different gliflozin or control schemes the use of multiple treatment NMA was prespecified. Calculations were performed in the R statistical software package version 4.1.3 (8, 9) using the packages “meta 5.2-0” and “netmeta 2.1-0.” A *p*-value < 0.05 was considered to represent statistical significance.

The risk ratio (RR) and its standard error were calculated from data from individual studies and entered into an NMA model. Direct and indirect comparisons were pooled in a frequentist approach, multiple treatment NMA that allows for multiple relationships to be integrated into the analysis accounting for the correlating treatment effects. Within this model, nodes were defined as the individual study arms and combined effect estimates with their 95% confidence interval (CI) were then calculated for each edge combined in a random-effect network.

Cochran’s Q statistics and its corresponding *p*-value measuring the heterogeneity as well as values of I2 representing the amount of inconsistency in the network were also calculated. In the case of the latter values, I2 < 25%, I2 > 25% but < 50% and I2 > 50% indicated a low, moderate, or substantial heterogeneity, respectively (10).

To assess publication bias, a comparison-adjusted funnel plot, an extension of the common funnel plot in cases of multiple treatment comparisons was used with Eggers’ test results in support (11).

## Results

**FIGURE 1:**
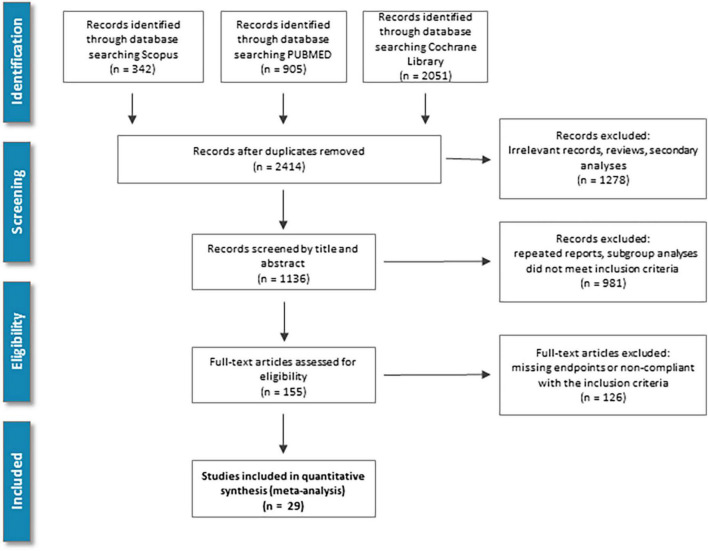
Study screening and selection flow: Overview of study screening and selection process according to PRISMA guidelines.

**FIGURE 2:**
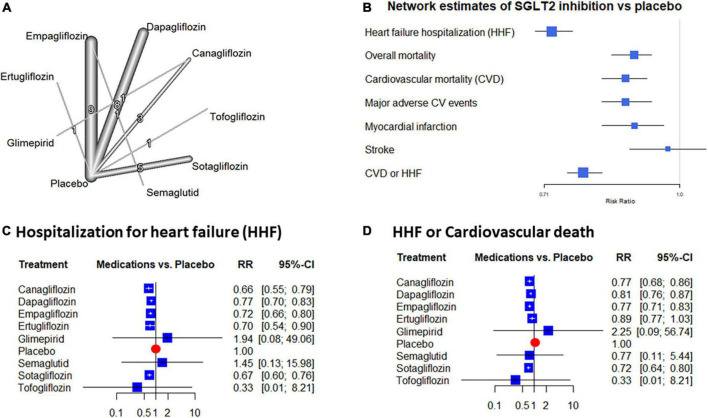
Evidence network and network estimates of the placebo compared effects: The network graph shows the individual treatment arms of the included trials depicting treatments as nodes and direct comparisons as edges. The numbers and the thickness of the edges show the direct comparisons performed **(A)**. **(B)** Depicts the estimates of gliflozin treatment compared to placebo extracted from the random effects network. **(C,D)** Show forest plots of heart failure hospitalization (HHF) and the HHF + cardiovascular mortality (CVD) endpoints. The relative risk (RR) values are presented as squares with whiskers showing their 95% confidence interval.

Our literature review resulted in the identification of 29 RCTs that met the predefined selection criteria and contained sufficient data for statistical analysis. The trials included data from 88,418 patients (Figure 1). Sixteen trials included only patients with known diabetes mellitus, and 4 trials recruited cases with known chronic kidney disease (CKD). HF patients were recruited in 16 trials. Among these 9 were performed with the participation of HF patients with known reduced ejection fraction (HFrEF), 3 with preserved ejection fraction (HFpEF), and 4 patients with acutely decompensated HF (Table 1 and Figure 2A). The selected studies were placebo-controlled randomized trials except for 2 cases where the control group received glimepiride or semaglutid medications (12, 13). None of the trials tested direct comparison between different gliflozin agents. Empagliflozin was assessed in 10, dapagliflozin in 8, sotagliflozin in 5, canagliflozin in 4, while tofogliflozin, and ertugliflozin in a single trial. Due to the different trial designs, the follow-up time of the included trials in our NMA varied between 1 and 50 months (Table 1 and Supplementary Table 1).

**TABLE 1:** Clinical characteristics of the included patient populations.

Trial	References	Country	Patient cohort	Treatment	No. of Patients	Median follow-up (months)	Primary outcome	Inclusion criteria	Main exclusion criteria
CANDLE	Tanaka et al. (13)	Japan	T2D and CHF	Canagliflozin (100 mg) vs. Glimepiride (0.5 mg)	245	6	Change in NT-proBNP	• T2D • NYHA I–III	• Severe renal dysfunction • NYHA class IV • Revascularization within 3 months
CANVAS	Neal et al. (24)	Multiple	T2D	Canagliflozin (100 mg, or 300 mg) vs. Placebo	10,142	28.8	MACE	• T2D • > 2 risk factors or history of ASCVD • eGFR > 30	• History of dialysis or renal transplant. • Recent ASCVD accident or revascularization • NYHA IV
Charaya K. et al.	Charaya et al. (33)	Russian Federation	Acute HF	Dapagliflozin (10 mg) vs. Placebo	102	1	Deterioration of renal function	• Acute HF	• Shock, mechanical ventilation • eGFR < 30 acute HF triggered by MI or PE
Cherney D. et al.	Cherney et al. (34)	Multiple	T2D and CKD4	Sotagliflozin (200 mg or 400 mg) vs. Placebo	277	12	change HbA1c	• T2D • HbA1c 7–11% • eGFR 15 –30	• Hypotension, immunosuppressive therapy, or dialysis
CHIEF-HF	Spertus et al. (35)	USA	HF	Canagliflozin (100 mg) vs. Placebo	448	3	change in KCCQ score	• HF • KCCQ > 40 and < 80	• History of ketoacidosis • Acute HF within 4 weeks • CKD > 4
CREDENCE	Perkovic et al. (29)	Multiple	T2D and CKD	Canagliflozin (100 mg) vs. Placebo	4,401	31.44	Worsening of CKD	• T2D, HbA1c 6.5–12.0% • GFR 30–90 with albuminuria	• Non-diabetic kidney disease • Immunosuppression treatment • Dialysis or kidney Tx
DAPA-CKD	Heerspink et al. (36)	Multiple	CKD	Dapagliflozin (10 mg) vs. Placebo	4304	28.8	Worsening of CKD	• GFR 25-90 with albuminuria	• T1D, • Immunotherapy within 6 months
DAPA-HF	McMurray et al. (4)	Multiple	HFrEF	Dapagliflozin (10 mg) vs. Placebo	4,744	18.2	worsening HF failure or CV death	• NYHA II-IV and LVEF ≤ 40% • NT-proBNP > 600	• T1D • Hypotension • eGFR < 30
DECLARE-TIMI 58	Wiviott et al. (27)	Multiple	T2D	Dapagliflozin (10 mg) vs. Placebo	17,160	50.4	MACE	• T2D, HbA1c 6.5–12.0% • Multiple ASCVD risk factors • CRCl > 60 ml	• ACS
DEFINE-HF	Nassif et al. (37)	USA	HFrEF	Dapagliflozin (10 mg) vs. Placebo	263	3	Improvement in health status or NT-proBNP	• NYHA II-III and LVEF < 40% • GFR ≥ 30 • BNP ≥ 100 and/or NT pro-BNP ≥ 400	• ACS • Planned CV revascularization
DELIVER	Solomon et al. (3)	Multiple	HFpEF	Dapagliflozin (10 mg) vs. Placebo	6,263	27.6	Worsening HF, or CV	• LVEF > 40% • NT-pro BNP ≥ 300	• Type 1 diabetes mellitus • eGFR < 25 • Unstable ASCVD or planned revascularization
EMPA-HEART Cardiolink-6	Verma et al. (38)	Canada	T2D and CAD	Empagliflozin (10 mg) vs. Placebo	97	6	Change in LV mass index	• T2D: HbA1C ≥ 6.5 and ≤ 10% • Established ASCVD	• eGFR < 60 • LVEF < 30% • NYHA Class IV • Unstable ASCVD
EMPA-REG OUTCOME	Zinman et al. (18)	Multiple	T2D	Empagliflozin (10 mg or 25 mg) vs. Placebo	7,020	37.2	MACE	• T2D: HgA1C 7.0–9.0% • BMI < 45 • eGFR > 30 • Established ASCVD	• Planned revascularization • Unstable ASCVD
EMPA-RESPONSE-AHF	Damman et al. (39)	Netherlands	Acute HF	Empagliflozin (10 mg) vs. Placebo	80	1	Clinical stabilization, change in NT-proBNP	• Acute HF • BNP ≥ 350 • eGFR: ≥ 30	• Type 1 diabetes mellitus • ACS • Planned revascularization
EMPA-TROPISM	Santos-Gallego et al. (40)	USA	HFrEF	Empagliflozin (10 mg) vs. Placebo	84	6	Change in LV parameters	• NYHA II, III and LVEF < 50%	• History of diabetes • ACS • GFR < 30
EMPEROR-Preserved	Anker et al. (2)	Multiple	HFpEF	Empagliflozin (10 mg) vs. Placebo	5,988	26.2	Hospitalization for HF or CV death	• NYHA II–IV and LVEF > 40% • NT-proBNP > 300	• ACS • eGFR < 20
EMPEROR-Reduced	Packer et al. (1)	Multiple	HFrEF	Empagliflozin (10 mg) vs. Placebo	3,730	16	Hospitalization for HF or CV death	• NYHA II–IV and LVEF < 40% • Elevated NT-proBNP	• ACS • eGFR < 20
EMPIRE HF	Jensen et al. (41)	Denmark	HFrEF	Empagliflozin (10 mg) vs. Placebo	190	3	Change of NT-proBNP	• NYHA I–III and LVEF < 40% • eGFR > 30	• NYHA IV • Recent hospitalization for HF or hypoglycemia
EMPULSE	Voors et al. (42)	Multiple	Acute HF	Empagliflozin (10 mg) vs. Placebo	530	3	All-cause death, HF events, change in KCCQ-TSS	• Acute HF • NT-proBNP > 1,600	• Cardiogenic shock • PE, stroke, or ACS • eGFR < 20 ml • Requiring dialysis
inTANDEM1	Buse et al. (43)	North America	T1D	Sotagliflozin (200 mg or 400 mg) vs. Placebo	793	12	HbA1c change	• T1D: HbA1c 7.0- 11.0%	• eGFR < 45 • Immunosuppressive therapy, or dialysis • NYHA III or IV HF • Significant ASCVD
inTANDEM2	Danne et al. (44)	Multiple	T1D	Sotagliflozin (200 mg or 400 mg) vs. Placebo	258	12	HbA1c change	• T1D: HbA1c 7.0–11.0%	• eGFR < 45 • NYHA III or IV HF • Significant ASCVD
PIONEER 2	Rodbard et al. (12)	Multiple	T2D	Empagliflozin (25 mg) vs. Semaglutid (14 mg)	422	12	HbA1c change	• HbA1c 7.0–10.5%	• eGFR: < 60 • Unstable ASCVD • NYHA IV
PRESERVED-HF	Nassif et al. (45)	USA	HFpEF	Dapagliflozin (10 mg) vs. Placebo	324	3	KCCQ-CS	• NYHA II-IV and EF ≥ 45% • NT-proBNP ≥ 225 or BNP ≥ 75	• T1D • eGFR < 20 • ACS or revascularization within 30 days
REFORM	Singh et al. (46)	UK	T2D and HFrEF	Dapagliflozin (10 mg) vs. Placebo	56	12	Change in LV end-systolic volume	• T2D • HFrEF: NYHA I–III, and LVEF < 45% • eGFR of > 45	• HbA1c < 6.0%.
SCORED	Bhatt et al. (28)	Multiple	T2D and CKD	Sotagliflozin (200 mg to 400 mg) vs. Placebo	10,584	16	MACE and hospitalization for HF or CV death	• T2D: HbA1c > 7.0% • eGFR: 25–60 ml • Risks for ASCVD	• End-stage HF • Planned revascularization
SOLOIST-WHF	Bhatt et al. (47)	Multiple	T2D and HFrEF	Sotagliflozin (200 mg to 400 mg) vs. Placebo	1222	9.2	Hospitalization for HF or CV death	• T2D • HF	• ACS • End-stage HF • eGFR < 30
SUGAR-DM-HF	Lee et al. (21)	UK	T2D and HFrEF	Empagliflozin (10 mg) vs. Placebo	105	9	LV parameters	• T2D: HbA1c ≤ 11% • NYHA II-IV and LVEF ≤ 40%	• eGFR < 30 • Unstable ASCVD
UTOPIA	Katakami et al. (32)	Japan	T2D	Tofogliflozin (20 mg) vs. Placebo	340	3	Changes in carotid ultrasound	• T2D: HbA1c ≥ 6% but < 9%	• History of ASCVD • eGFR of < 30 • NYHA > III
VERTIS CV	Cannon et al. (31)	Multiple	T2D	Ertugliflozin (5 mg or 15 mg) vs. Placebo	8,246	42	MACE	• T2D: HbA1c 7.0–10.5% • Established ASCVD	• Planned revascularization • NYHA IV • GFR < 30

ACS, acute coronary syndrome; ASCVD, atherosclerotic cardiovascular disease; CV, cardiovascular; BMI, Body mass index (kg/m2); BNP, brain natriuretic peptide (pg/ml); CHF, chronic heart failure; CKD, chronic kidney disease (stage); CrCL, creatinine clearance (ml/min); GFR, glomerular filtration rate (ml per minute per 1.73 m2 of body-surface area); HbA1c, glycated hemoglobin level; HF, heart failure; HFpEF, heart failure with preserved ejection fraction; HFrEF, heart failure with reduced ejection fraction; KCCQ, Kansas City Cardiomyopathy Questionnaire score; LV, left ventricular; LVEF, left ventricular ejection fraction; MACE, major adverse cardiovascular events; MI, myocardial infarction; NT-proBNP, N terminal pro-brain natriuretic peptide (pg/ml); NYHA, New York Heart Association functional class; T2D, diabetes mellitus type 2; T1D, diabetes mellitus type 1; PE, pulmonary embolism; Tx, transplantation.

Quality assessment of the included studies showed no major risk of bias and the comparison-adjusted funnel plot analyses did not detect important signals suggesting important publication bias (Supplementary Figures 1, 2).

Compared to placebo, all gliflozin treatments reduced the rate of HHF. This benefit ranged from a 23 to 54% reduction of risk with all SGLT2 inhibitors except with tofogliflozin. In this latter case due to the large CI, the risk reduction did not reach the level of statistical significance. The inconsistency was low to moderate in these data (tau2 = 0.007; tau = 0.835; I2 = 27.3% [0.0%; 56.8%]) without significant total or within design heterogeneity (*p* = 0.90, both). Based on the network estimates SGLT2 inhibition offers a significant 28% reduction of HHF risk (RR: 0.72 [0.69; 0.76]) (Figure 2).

Following most of the individual trials, the composite endpoint of HHF + CVD was beneficially affected by SGLT2 inhibition. Analyses of this endpoint reflected a significant 11–28% risk reduction with canagliflozin, dapagliflozin, empagliflozin, and sotagliflozin. In the case of ertugliflozin and tofogliflozin the reduction was not significant but the gliflozins altogether resulted in a 22% significant risk reduction (RR: 0.78 [0.75; 0.82]) (Figure 2).

**FIGURE 3:**
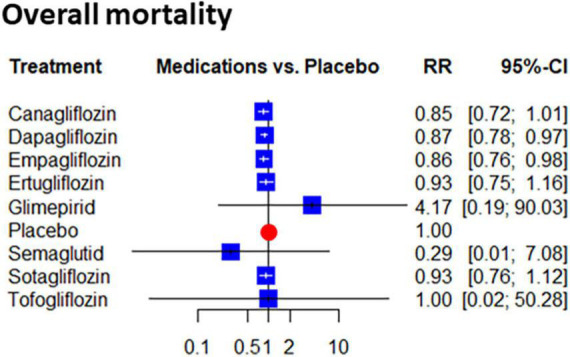
Network estimates of placebo compared effect on the risk of the overall mortality: Forest plots show the relative risk (RR) values and their 95% confidence intervals of individual treatments as estimated on mortality.

Two gliflozins significantly reduced overall mortality. This benefit reached 13% with dapagliflozin and 14% with empagliflozin (RR: 0.87 [0.78; 0.97], and RR: 0.86 [0.76; 0.98], respectively). The effect of the other gliflozins did not reach the level of statistical significance. However, risk reduction estimates were in a similar range except for tofogliflozin where a neutral effect was estimated. The inconsistency reached a moderate level (tau2 = 0.007; tau = 0.0835; I2 = 27.3% [0.0%; 56.8%]) but total and within design heterogeneities were non-significant (*p* = 0.12, both). Altogether the mortality reduction with SGLT2 inhibitors was significant compared to placebo (RR: 0.89 [0.84; 0.93], the *p*-value for heterogeneity = 0.26) (Figure 3).

**FIGURE 4:**
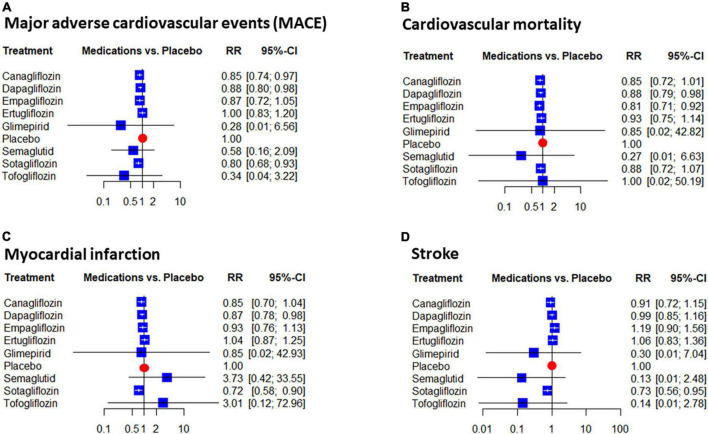
Network estimates of placebo compared the effect on the risk of major adverse cardiovascular events (MACE): Forest plots show the relative risk (RR) values and their 95% confidence intervals of individual treatments as estimated on MACE **(A)** and its components: Cardiovascular mortality **(B)**, myocardial infarction **(C)**, stroke **(D)**.

A significant reduction was seen in the risk estimates of CVD with dapagliflozin (RR: 0.88 [0.79; 0.98] and empagliflozin (RR: 0.81 [0.71; 0.92]). Similarly, to the overall mortality, the effect of tofogliflozin was neutral, but the gliflozins as a group significantly reduced the risk of this endpoint (RR: 0.87 [0.82; 0.92]). The data showed a low level of inconsistency in this regard (I2 = 0.0% [0.0%; 42.5%], pheterogeneity = 0.52) (Figure 4).

Three drugs, canagliflozin, dapagliflozin, and sotagliflozin significantly reduced the rate of MACE. The rate of this benefit ranged from 12 to 20% (Figure 4). The risk reduction of MACE with gliflozins compared to placebo was also significant at 13% (RR: 0.87 [0.82; 0.93], I2 = 25.5% [0.0%; 55.8%]), pheterogeneity = 0.13). Among the components of MACE, MI, and stroke were significantly affected by sotagliflozin (RR: 0.72 [0.58; 0.90] and RR: 0.73 [0.56; 0.95], respectively) and dapagliflozin lowered MI risk compared to placebo (RR: 0.87 [0.78; 0.98]). The analyses of the risk of MI also supported a significant benefit with gliflozins (RR: 0.89 [0.82; 0.96]) (Figures 4C,D).

Gliflozin-to-gliflozin comparisons in our network supported the consistency of the benefits of gliflozin treatment. In the analyses of HHF, mortality, or CVD no significant differences were detected. Sotagliflozin appeared to be more effective regarding HHF + CVD, stroke, and MI compared to ertugliflozin, in HHF + CVD and stroke compared to dapagliflozin, and in stroke endpoint compared to empagliflozin (Table 2). In the subgroup analysis, we have to highlight that the gliflozins showed remarkable effectiveness compared to placebo on the whole spectrum of the included trials. Subgroup analyses further supported the consistency of the data regarding the benefits of gliflozin treatment. Gliflozin therapy showed significant benefits in terms of HHF + CVD, MACE, and mortality among patients with diabetes, CKD, or HF (Table 3). Sex-specific subgroup analyses showed no clear signal for influence (Supplementary Figure 3).

**TABLE 2:** League table of the indirect gliflozin to gliflozin comparisons.

	Endpoint	Canagliflozin	Dapagliflozin	Empagliflozin	Ertugliflozin	Sotagliflozin	Placebo
Canagliflozin	Stroke	**Canagliflozin**	.	.	.	.	0.91 (0.72; 1.15)
	MI		.	.	.	.	0.85 (0.70; 1.04)
	HHF + CVD		.	.	.	.	0.77 (0.68; 0.86)*
Dapagliflozin	Stroke	0.91 (0.69; 1.21)	**Dapagliflozin**	.	.	.	0.99 (0.85; 1.16)
	MI	0.98 (0.78; 1.23)		.	.	.	0.87 (0.78; 0.98)*
	HHF + CVD	0.94 (0.83; 1.08)		.	.	.	0.81 (0.76; 0.87)*
Empagliflozin	Stroke	0.76 (0.53; 1.10)	0.84 (0.61; 1.15)	**Empagliflozin**	.	.	1.19 (0.90; 1.56)
	MI	0.92 (0.69; 1.21)	0.94 (0.75; 1.18)		.	.	0.93 (0.76; 1.13)
	HHF + CVD	1.00 (0.87; 1.15)	1.06 (0.96; 1.17)		.	.	0.77 (0.71; 0.83)*
Ertugliflozin	Stroke	0.85 (0.61; 1.20)	0.94 (0.70; 1.26)	1.12 (0.77; 1.62)	**Ertugliflozin**	.	1.06 (0.83; 1.36)
	MI	0.82 (0.62; 1.07)	0.84 (0.67; 1.04)	0.89 (0.68; 1.17)		.	1.04 (0.87; 1.25)
	HHF + CVD	0.86 (0.72; 1.04)	0.91 (0.78; 1.07)	0.86 (0.73; 1.02)		.	0.89 (0.77; 1.03)
Sotagliflozin	Stroke	1.24 (0.87; 1.77)	1.36 (1.00; 1.85)*	1.63 (1.11; 2.38)*	1.45 (1.01; 2.09)*	**Sotagliflozin**	0.73 (0.56; 0.95)*
	MI	1.18 (0.88; 1.58)	1.21 (0.94; 1.55)	1.29 (0.96; 1.73)	1.44 (1.08; 1.92)*		0.72 (0.58; 0.90)*
	HHF + CVD	1.07 (0.91; 1.25)	1.13 (1.00; 1.28)*	1.07 (0.94; 1.22)	1.24 (1.03; 1.49)*		0.72 (0.64; 0.80)*
Tofogliflozin	Stroke	6.32 (0.32; 123.50)	6.93 (0.36; 134.66)	8.27 (0.42; 162.10)	7.40 (0.38; 144.74)	5.09 (0.26; 99.66)	0.14 (0.01; 2.78)
	MI	0.28 (0.01; 6.90)	0.29 (0.01; 7.04)	0.31 (0.01; 7.53)	0.35 (0.01; 8.44)	0.24 (0.01; 5.86)	3.01 (0.12; 72.96)
	HHF + CVD	2.29 (0.09; 56.30)	2.42 (0.10; 59.49)	2.29 (0.09; 56.28)	2.65 (0.11; 65.26)	2.14 (0.09; 52.65)	0.33 (0.01; 8.21)

The lower left half of the table shows the indirect assessment network estimates of SGLT2 inhibitor comparisons. The upper right part shows the meta-analysis result of the direct placebo to gliflozin comparisons. Data are shown as relative risk and 95% confidence intervals. *Marks significant effect estimates. MI, myocardial infarction; HHF + CVD, hospitalization for heart failure or cardiovascular death.

**TABLE 3:** Subgroup analyses.

Subgroup	Nr. of trials	Nr. of participants	Endpoint	Canagliflozin	Dapagliflozin	Empagliflozin	Ertugliflozin	Sotagliflozin	Tofogliflozin	SGLT2i
Diabetes trial (100% diabetes)	16	61,368	CVD + HHF	0.76 (0.65; 0.89)*	0.83 (0.69; 1.00)*	0.68 (0.55; 0.85)*	0.89 (0.72; 1.09)	0.71 (0.61; 0.82)*	0.33 (0.01; 8.23)	0.76 (0.70; 0.83)*
			MACE	0.84 (0.67; 1.06)	0.91 (0.68; 1.24)	0.89 (0.65; 1.23)	1.00 (0.73; 1.37)	0.78 (0.62; 0.99)*	0.34 (0.03; 3.27)	0.87 (0.80; 0.95)*
			Mortality	0.86 (0.77; 0.97)*	0.92 (0.82; 1.04)	0.70 (0.58; 0.83)*	0.93 (0.80; 1.08)	0.95 (0.82; 1.10)	1.00 (0.02; 50.11)	0.87 (0.81; 0.95)*
Predominantly diabetic patients (>50%)	19	66,259	CVD + HHF	0.76 (0.67; 0.87)*	0.81 (0.71; 0.92)*	0.68 (0.56; 0.82)*	0.89 (0.75; 1.05)	0.72 (0.63; 0.81)*	0.33 (0.01; 8.22)	0.76 (0.71; 0.82)*
			MACE	0.84 (0.69; 1.03)	0.87 (0.71; 1.07)	0.89 (0.67; 1.18)	1.00 (0.75; 1.32)	0.79 (0.63; 0.98)*	0.34 (0.03; 3.25)	0.87 (0.80; 0.94)*
			Mortality	0.86 (0.74; 1.00)*	0.85 (0.73; 0.98)*	0.70 (0.56; 0.87)*	0.93 (0.77; 1.13)	0.93 (0.78; 1.12)	1.00 (0.02; 50.22)	0.86 (0.79; 0.93)*
CKD trial	4	19,566	CVD + HHF	0.71 (0.29; 1.75)	0.72 (0.29; 1.82)	NA	NA	0.58 (0.27; 1.24)	NA	0.72 (0.64; 0.80)*
			MACE	0.81 (0.17; 3.72)	0.78 (0.17; 3.61)	NA	NA	0.47 (0.15; 1.52)	NA	0.77 (0.66; 0.89)*
			Mortality	0.83 (0.55; 1.28)	0.69 (0.44; 1.09)	NA	NA	0.94 (0.63; 1.39)	NA	0.84 (0.69; 1.02)
Predominantly CKD patients (>50%)**	6	21,318	CVD + HHF	0.71 (0.58; 0.86)*	0.72 (0.56; 0.94)*	0.69 (0.46; 1.05)	NA	0.72 (0.63; 0.81)*	NA	0.72 (0.66; 0.78)*
			MACE	0.81 (0.47; 1.37)	0.78 (0.44; 1.35)	NA	NA	0.75 (0.52; 1.09)	NA	0.80 (0.69; 0.92)*
			Mortality	0.83 (0.69; 1.02)	0.69 (0.54; 0.89)*	0.50 (0.25; 1.01)	NA	0.96 (0.82; 1.11)	NA	0.82 (0.70; 0.97)*
Heart failure trial	16	24,374	CVD + HHF	0.98 (0.29; 3.35)	0.81 (0.74; 0.88)*	0.79 (0.72; 0.86)*	NA	0.72 (0.62; 0.83)*	NA	0.79 (0.75; 0.83)*
			MACE	NA	0.88 (0.76; 1.01)	1.12 (0.31; 4.00)	NA	0.98 (0.71; 1.33)	NA	0.89 (0.81; 0.98)*
			Mortality	0.49 (0.09; 2.65)	0.90 (0.82; 0.98)*	0.95 (0.87; 1.05)	NA	0.86 (0.63; 1.18)	NA	0.92 (0.86; 0.98)*
HFrEF	9	10,474	CVD + HHF	NA	0.65 (0.26; 1.64)	0.77 (0.35; 1.67)	NA	0.72 (0.26; 1.97)	NA	0.76 (0.69; 0.83)*
			MACE	NA	0.86 (0.45; 1.62)	1.11 (0.29; 4.24)	NA	0.98 (0.43; 2.22)	NA	0.90 (0.75; 1.08)
			Mortality	NA	0.83 (0.72; 0.97)*	0.93 (0.80; 1.10)	NA	0.86 (0.63; 1.18)	NA	0.88 (0.79; 0.97)*
HFpEF	3	12,575	CVD + HHF	NA	0.84 (0.76; 0.94)*	0.81 (0.72; 0.91)*	NA	NA	NA	0.83 (0.76; 0.90)*
			MACE	NA	0.89 (0.77; 1.03)	NA	NA	NA	NA	0.89 (0.77; 1.03)
			Mortality	NA	0.94 (0.84; 1.06)	0.99 (0.87; 1.12)	NA	NA	NA	0.96 (0.89; 1.05)
Acute heart failure	4	1,934	CVD + HHF	NA	NA	0.65 (0.44; 0.96)*	NA	0.72 (0.63; 0.82)*	NA	0.71 (0.62; 0.81)*
			MACE	NA	NA	0.32 (0.03; 2.92)	NA	0.98 (0.74; 1.28)	NA	0.96 (0.73; 1.26)
			Mortality	NA	0.78 (0.36; 1.69)	0.48 (0.25; 0.94)*	NA	0.86 (0.63; 1.18)	NA	0.78 (0.60; 1.01)
Long-term (>6 months)	18	85,715	CVD + HHF	0.76 (0.67; 0.87)*	0.81 (0.75; 0.87)*	0.77 (0.70; 0.84)*	0.89 (0.75; 1.05)	0.72 (0.63; 0.81)*	NA	0.78 (0.74; 0.82)*
			MACE	0.85 (0.72; 0.99)*	0.88 (0.78; 0.98)*	0.88 (0.70; 1.10)	1.00 (0.80; 1.24)	0.79 (0.67; 0.95)*	NA	0.87 (0.81; 0.93)*
			Mortality	0.86 (0.71; 1.04)	0.86 (0.76; 0.98)*	0.88 (0.76; 1.02)	0.93 (0.72; 1.20)	0.92 (0.74; 1.13)	NA	0.88 (0.83; 0.94)*

The table depicts the network effect estimates of the different SGLT2 inhibitors and SGLT2 inhibitors as a group compared to placebo in the predefined subgroups. Data are presented as risk ratio (95% confidence interval). *Marks significant effect estimates. **The subgroup is defined as studies with more than 50% of patients with eGFR < 60 ml/min/1,73 m^2^. SGLT2i, sodium-glucose transporter 2 inhibitors; CVD + HHF, cardiovascular mortality and hospitalization for heart failure; MACE, major adverse cardiac events; CKD, chronic kidney disease; HFpEF, heart failure with preserved left ventricular ejection fraction; HFrEF, heart failure with reduced left ventricular ejection fraction.

## Discussion

HF is one of the major contributors to CV morbidity and mortality in patients with or without diabetes (14, 15). In the recent past, a multitude of clinical trial data were published related to the management of HF. These included patients diagnosed with a wide symptomatic or left ventricular (LV) EF range of the HF spectrum. The trials included cases with symptomatic and/or acutely decompensated HF as well as populations with an elevated risk of HF development. In addition to HF associated with an HFrEF where multiple treatment alternatives may be used lately also positive trials were published widening the painfully narrow options in the case of HFpEF (1, 3).

Among other promising therapeutic options inhibition of the SGLT2 with gliflozins was figured as one of the breakthroughs showing benefits on top of the established strategies (14). The present meta-analysis combining CV outcome data from a broad summary of RCTs of gliflozins supports the efficacy and safety of this pharmacologic approach. We found that benefits were consistent in the overall analyses and relevant subgroups without a signal of major gliflozin-to-gliflozin differences. Including a wide range of RCTs and an intended comprehensive analysis of data on gliflozin treatment, our results support a favorable group effect of SGLT2 inhibition in the HF spectrum.

Previously SGLT2 inhibitors were investigated extensively among patients with diabetes. Impacts on the macro and microvasculature in T2DM considerably increase the risk of CV adverse events with a consequent increase in mortality and morbidity. The therapeutic strategies used in T2DM may have an impact on the occurrence of CV events. Even though there are several drugs available for the treatment of T2DM that are proven to be beneficial for glycemic control, they mostly failed behind the expectations in terms of CV risk reduction (16). Nonetheless, a breakthrough with glucagon-like peptide 1 receptor agonist (GLP1-1RA) and SGLT2 inhibitors was seen. While the earlier data showed at best neutral results, the CV outcome data of these two groups suggested effectiveness in patients with CV disease or CV risk factors (17). Among the earliest trials in the EMPA-REG OUTCOME trial, empagliflozin significantly reduced the incidence of CVD, HF hospitalizations, and all-cause mortality (18).

The complex interaction among mechanisms including neurohormonal activation, volume regulation as well as that atherosclerotic progression offers multiple mechanisms that orchestrate the witnessed efficacy of gliflozins. The mechanisms may be related to altering diuresis and natriuresis, the consequential afterload reduction, better myocardial metabolism as well as improvement of vascular function and structure. Furthermore, SGLT2 inhibitors may have a favorable effect on cardiac remodeling, and the reduction in N-terminal pro-B-type natriuretic peptide observed with gliflozins is consistent with these mechanisms (1, 4, 19). Additionally, benefits may be associated with but not restricted to the SGLT2 inhibition itself. These may also be explained by additional effects on adipokine production as well as alterations in the myocardial Na^+^/H^+^ exchange (19). Gliflozins have also been anticipated to lower cardiac oxidative stress and inflammation through the promotion of the actions of sirtuin-1. Actions effective *via* the downregulation of hypoxia-induced signaling also require further confirmation (20). Mechanisms at the level of LV function are also targeted to further studies. Empagliflozin led to favorable reverse LV remodeling in patients with HFrEF and T2DM or prediabetes. However, an increase in LV EF with empagliflozin treatment was not reflected in all studies (21). The fact that the complex interaction of mechanisms was evident in such a wide variety of conditions supports the assumption that more than one principal process orchestrates together and some of them may have variable importance according to the clinical scenario.

The most common side effect of SGLT2 inhibitors is related to their mechanism of action. As they inhibit glucose reabsorption through the kidneys, it is excreted in the urine, and it may increase the risk of recurrent genital and urinary tract bacterial or mycotic infections as well. It is the most common adverse effect; the incidence rate is around 5%. Genital infections cause only mild problems, but they may contribute to weak adherence. Furthermore, diabetes has an effect compromising surface immunity, and patients with diabetes may have a 24% increased risk of genital infections (22).

In HFrEF, maladaptive neurohormonal activation is considered to be the primary driver of the disease and symptom development (15). Treatments interrupting this vicious circle are effectively reducing and hampering progression. Importantly, gliflozins were applied on top of the combinations composed of complex neurohormonal aimed therapies, including state-of-the-art regimes of angiotensin-converting enzyme inhibitors (ACEI), angiotensin receptor blockers (ARB), mineralocorticoid receptor antagonists (MRA), and β-blockers. According to the latest ESC guideline ACEIs, β-blockers, and MRAs are the fundamentals for patients with HFrEF, these medications are necessary in all cases. SGLT2 inhibitors are recommended to be added to this base therapy regardless of diabetes status. It is important to emphasize that, according to the latest guideline instead of a stepwise approach SGLT2 inhibitors can be started early in HFrEF (15).

Among the high number of gliflozins two drugs, dapagliflozin and empagliflozin were studied most extensively. The finding of our analysis, support that also the other drugs from the SGLT2 inhibitor class share these beneficial properties. The recent European guideline also suggests the use of canagliflozin, ertugliflozin, and sotagliflozin (15). Detailed descriptions reach beyond the scope of this work. We refer to a recent review where the history of the different SGLT2 inhibitors is reviewed in detail (23).

Recent studies question whether with SGLT2 inhibitors major alterations in myocardial blood flow or extracellular volume would be detectable (21). However, data with regard to the reduction of ischemic events and results of diabetic CV outcome trials support assumptions that the effects of lowering blood sugar levels and improving metabolic balance may alleviate the progression of vascular disease (18, 24). In line with these, in our comprehensive analysis of SGLT2 inhibition, which reflected an improvement in MACE, and its components, we found that these results were in close association with the magnitude of HHF and the mortality data. In the light of data supporting consistent, similar prognostic benefits in patients with or without diabetes the improvements of glucose metabolisms may be an effect with magnitude considerably differing according to the functional status, risk profile, and also the treatment duration of the individual patient (25).

At the pivotal trials both with dapagliflozin and empagliflozin the composite endpoint of CVD and HHF was reduced in patients with HFrEF. CVD and overall mortality, however, were significantly reduced with dapagliflozin but remained unaffected with empagliflozin (1, 4). Similarly, to the latter, both drugs reduced HHF + CVD in HFpEF, but neither mortality endpoints (2, 26). In our analyses, however, the important all-cause mortality and CVD benefit were characterized both in the overall analyses and in most subgroups. Together with the lack of a signal for major gliflozin to gliflozin differences, this explains that the individual studies were underpowered to reliably detect this effect but also underlines the fact that the HF benefits of gliflozins do not come with a price compromising the life expectancy of the patients.

The DECLARE-TIMI 58 trial showed a 7% reduction of the composite ischemic endpoints, a benefit that did not reach the level of statistical significance (RR: 0.93 [0.84; 1.03]) (27). The NMA of the gliflozin trials extended the earlier observations of MACE reduction associated with canagliflozin or sotagliflozin that a similar range of benefits also associated with dapagliflozin therapy (24, 28, 29). It is of note that in the subgroup analyses SGLT2 inhibitors showed a significant 11% reduction of MACE risk, a difference that also appeared in the HFrEF and HFpEF subgroups but due to the wider confidence intervals remained non-significant.

Importantly, the field of HFpEF in the past resulted in a high number of negative trials where gliflozins mark an important breakthrough. Besides the beneficial effect among patients with HF or with a substantial risk of developing HF due to compromised EF, gliflozins showed important improvement among HFpEF. Before the advent of gliflozins in HFpEF, our treatment algorithms concentrated on the risk factors of HFpEF development, and medical therapy was restricted to diuretics for symptom relief, together with antihypertensive treatment using ACEIs, β-blockers, and ARBs, with the latter a potential to reduce HHF (14, 15). Compared with placebo, empagliflozin, and dapagliflozin regardless of diabetes status reduced the risk of the composite of HHF + CVD. This makes SGLT2 inhibition the first and only therapy to meet this milestone.

The subpopulation most affected by immediate hemodynamic and diuretic effects is composed of patients hospitalized in an event of acute HF. Trials testing SGLT2 inhibitors in this field included a considerably lower number of cases but did not find a major risk of gliflozins and showed an improved HF prognosis with gliflozins and a significant mortality benefit with empagliflozin. Our NMA confirmed the efficacy of the two SGLT2 inhibitors evaluated in this case, as the HHF + CVD endpoint was significantly reduced. When introduced during hospitalization or early post-discharge gliflozins reduced the risk of rehospitalization for HF and improved outcomes without the excess risk of adverse effects (30).

Trial designs of our times are based on meticulous power calculations and cost considerations. This resulted in the wide use of composite endpoint criteria, which were also used in our included studies, such as HHF + CVD and MACE. We consider our NMA’s limitation that none of the included studies used only single outcomes, therefore CV efficacy could also be evaluated based on composite endpoints. Nevertheless, in the era of composite endpoints mortality continues to play a prominent role, and it was evaluated in every included trial. As to our NMA’s limitation, we have to mention that we could only include a single trial of tofogliflozin or ertugliflozin, which lead to uncertainty of the effect estimation, reflected by the wider confidence intervals. As more than 8.000 patients were included in the VERTIS-CV trial with ertugliflozin the estimates are more reliable (31). However, only 340 patients were included in a study with tofogliflozin (32). According to the clinicaltrials.gov database currently, only one study is running that has a tofogliflozin active arm thus we do not expect that considerably more data will be available soon to improve this estimation. The indirect gliflozin to tofogliflozin comparisons did not show a signal for important differences, however, these should be interpreted cautiously. For further limitation, the included trials in our analysis consisted of patients with a wide range of glomerular filtration rates (GFR). The inclusion criteria of these trials permitted that the effectiveness of the tested SGLT2 inhibitors was proven in the case of patients with different GFRs. In the current analysis, we concentrated on CV endpoints and analyses of kidney-related endpoints or kidney function remained beyond our scope. In our subgroup analysis, we found that in patients with impaired renal function SGLT2 inhibitors significantly reduced the risk of HHF + CVD, MACE, and overall mortality endpoints. However, it is important to note that kidney function was not uniformly reported in most of the included trials, this impedes further analysis in this direction.

In summary, a review and NMA of current evidence demonstrated a highly statistically significant and clinically relevant risk reduction with gliflozin. SGLT2 inhibitors achieved on top of state-of-the-art treatment algorithms statistically significant, positive results for MACE and overall mortality. The safety profile of gliflozins characterizes a tolerable treatment option in patients with HF or with a high risk for HF development regardless of EF or diabetes status. These data endorse the recent European and AHA/ACC/HFSA recommendations treating SGLT2 inhibitors as foundational first-line therapies for HF and support the widening of these recommendations to all EF categories (14, 15).

## Data availability statement

The original contributions presented in this study are included in the article/Supplementary material, further inquiries can be directed to the corresponding author.

## Author contributions

DT and AK contributed to the study design, implementation of the statistical analysis, drafting, and revision of the manuscript. DT, AK, and MM contributed to the study design, the review of relevant articles, and the data extraction. PK, RL, RF, and OE contributed to the revision of the manuscript. AK was the guarantor, attested that all listed authors met authorship criteria and that no others meeting the criteria have been omitted, and declared that all authors gave their written permission to include their names in the article. All authors have read and approved the submitted manuscript, the manuscript has not been submitted elsewhere nor published elsewhere in whole or in part, except as an abstract.

## Abbreviations

ACEI: angiotensin-converting enzyme inhibitor
ARB: angiotensin receptor blocker
CI: confidence interval
CKD: chronic kidney disease
CV: cardiovascular
CVD: cardiovascular mortality
EF: ejection fraction
GFR: glomerular filtration rate
GLP1-1RA: glucagon-like peptide 1 receptor agonist
HF: heart failure
HFpEF: heart failure with preserved ejection fraction
HFrEF: heart failure with reduced ejection fraction
HHF: heart failure-related hospitalization
LV: left ventricular
MACE: major adverse cardiac events
MI: myocardial infarction
MRA: mineralocorticoid receptor antagonist
NMA: network meta-analysis
RCT: randomized controlled trial
RR: risk ratio
SGLT2: sodium-glucose transport protein 2
T2DM: type II diabetes mellitus.
