# Supplementary material for: Cardiovascular outcomes in patients treated with sodium-glucose transport protein 2 inhibitors, a network meta-analysis of randomized trials
Source: Front Cardiovasc Med. 2022 Dec 5;9:1041200. doi: 10.3389/fcvm.2022.1041200 (PMC9760750; doi:10.3389/fcvm.2022.1041200)
Supplement: Supplementary file 1 [file Data_Sheet_1.docx]

**Supplementary material**

**Table of contents**

[**Table S1. Clinical characteristics of the included patient populations** 2](#_Toc117939042)

[**Figure S1. Bias assessment** 4](#_Toc117939043)

[**Figure S2. Visual estimation of publication bias with funnel plots** 6](#_Toc117939044)

[**Figure S3. Summary of sex-specific subgroup analyses of the included trials** 8](#_Toc117939045)

[**Citations of the included trials** 10](#_Toc117939046)

# **Table S1. Clinical characteristics of the included patient populations**

| Trial acronym | Male (%) | Mean Age | BMI (kg/m2) | Hypertension (%) | Atherosclerosis (%) | CAD (%) | PAD (%) | DM (%) | Atrial fibrillation (%) | Heart failure (%) | Mean EF (%) | Ischemic HF (%) | Median NT-proBNP (pg/ml) | Mean eGFR | eGFR <60 mL/min per 1·73 m^2^ (%) |
| --- | --- | --- | --- | --- | --- | --- | --- | --- | --- | --- | --- | --- | --- | --- | --- |
| CANDLE | 74.7 | 68.6 | NA | 43.8 | NA | NA | NA | 100 | NA | 100 | 57 | 43 | NA | NA | NA |
| CANVAS Program | 64.2 | 63.3 | 32 | 89.9 | 65.6 | 56.4 | 20.8 | 100 | 6 | 14.4 | NA | NA | 91 | 76.5 | 20.1 |
| Charaya K. et al. | 57.8 | 73.4 | NA | 92 | NA | NA | NA | 30 | 53.9 | 100 | 45 | NA | 4706 | 51.6 | NA |
| Cherney D. et al. | 48.8 | 67.7 | 31.6 | NA | NA | NA | NA | 100 | NA | NA | NA | NA | NA | 24 | 100 |
| CHIEF-HF | 55.1 | 63.4 | NA | NA | NA | NA | NA | 27.9 | NA | 100 | NA | NA | NA | NA | NA |
| CREDENCE | 66.1 | 63 | 31.3 | 96.8 | 50.4 | 29.8 | 23.8 | 100 | 6.2 | 14.8 | NA | NA | NA | 56.2 | 58.9 |
| DAPA-CKD | 66.9 | 61.8 | 29.5 | 95.7 | 37.4 | NA | 7.6 | 67.5 | 5.3 | 11 | NA | NA | NA | 43.1 | 89.45 |
| DAPA-HF | 76.6 | 66.4 | 28.1 | NA | NA | NA | NA | 41.8 | 38.3 | 100 | 31 | 56.4 | 1437 | 65.8 | 40.6 |
| DECLARE-TIMI 58 | 62.6 | 64 | 32 | 93 | 40 | 33 | 6 | 100 | 6.5 | 10 | NA | 54.3 | 75 | 85.3 | 7.4 |
| DEFINE-HF | 73.4 | 61,3 | 30.6 | NA | NA | 70 | NA | 63.1 | 40.3 | 100 | 26 | 52.9 | 1136 | 69.5 | NA |
| DELIVER | 56.1 | 71.6 | 29.8 | 88.7 | 56.7 | 50.5 | 2.2 | 44.8 | 56.7 | 100 | 54.2 | NA | 1011 | 61 | 49 |
| EMPA-HEART Cardiolink-6 | 93 | 64 | 26.7 | 44 | 100 | 100 | 5 | 100 | 0 | 6 | 56 | NA | 106.5 | 87 | 0 |
| EMPA-REG OUTCOME | 71.5 | 63.1 | 30.6 | 95.1 | 1 | 75.6 | 20.7 | 100 | 5.5 | 10 | NA | NA | NA | 74 | 25.9 |
| EMPA-RESPONSE AHF | 67 | 76 | NA | 62 | NA | NA | NA | 33 | 71 | 100 | 36 | 28.5 | 5236 | 55 | NA |
| EMPA-TROPISM | 64 | 62 | 29.3 | 74 | NA | NA | NA | 0 | 21 | 100 | 36 | 50 | NA | 80 | NA |
| EMPEROR-Preserved | 55.4 | 71.9 | 29.8 | 90.6 | NA | NA | NA | 49.1 | 51.1 | 100 | 54 | 35.4 | 970 | 60.6 | 49.9 |
| EMPEROR-REDUCED | 76.1 | 66.85 | 27.9 | 72.3 | NA | NA | NA | 49.8 | 36.7 | 10.2 | 27 | 51.75 | 1906 | 62 | NA |
| EMPIRE-HF | 85 | 63.5 | 29 | NA | NA | 54.5 | NA | 18 | 36.8 | 100 | 30 | 51.1 | 594 | 73.5 | NA |
| EMPULSE | 66 | 71 | 28.7 | 80.4 | NA | NA | NA | 45.3 | 49.45 | 100 | 31 | NA | 3202 | 52 | 63.4 |
| inTANDEM 1 | 48.3 | 46.1 | 29.66 | NA | NA | NA | NA | 100 (Type 1) | NA | NA | NA | NA | NA | 87 | NA |
| inTANDEM 2 | 51.9 | 41.2 | 27.77 | NA | NA | NA | NA | 100 (Type 1) | NA | NA | NA | NA | NA | 91.66 | NA |
| PIONEER-2 | 50.5 | 58 | 32.8 | NA | NA | NA | NA | 100 | NA | NA | NA | NA | NA | 95 | NA |
| PRESERVED-HF | 43.2 | 70 | 34.8 | NA | NA | 19.5 | NA | 55.9 | 52.8 | 100 | 60 | 19.4 | 675 | 55 | NA |
| REFORM | 66.1 | 67.1 | 32.5 | 40 | NA | 51.8 | 16.1 | 100 | 39.3 | 100 | 45 | 53.6 | NA | 72 | NA |
| SCORED | 55.1 | 69 | 31.8 | NA | NA | NA | NA | 100 | NA | 31 | 60 | NA | 197 | 44.6 | 100 |
| SOLOIST-WHF | 66.3 | 70 | 30.8 | NA | NA | NA | NA | 100 | NA | 100 | 35 | NA | 1779 | 49.7 | 69.9 |
| SUGAR-DM-HF | 77 | 68.7 | 30.7 | 74 | NA | 74 | NA | 78.1 | 0 | 100 | 32 | NA | 466 | 67.3 | NA |
| UTOPIA | 58.4 | 61.1 | 27 | 56.95 | 0 | 0 | 0 | 100 | 0 | 0 | NA | 0 | 32.75 | 81.3 | NA |
| VERTIS-CV | 68.1 | 64.4 | 31.9 | 93.4 | NA | 75.9 | 18.7 | 100 | NA | 18 | NA | NA | NA | 75.9 | 21.9 |

**Abbreviations:** BMI: body mass index, CAD: coronary artery disease, PAD: peripheral atherosclerosis, DM: diabetes mellitus, EF: left ventricular ejection fraction, HF: heart failure, NT-proBNP: N-terminal pro-brain natriuretic peptide, eGFR: estimated glomerular filtration rate, NA: not added

# **Figure S1. Bias assessment**

The methodological quality of the included randomized control trials was assessed with the Cochrane Risk Bias tool.

**S1/A Bias assessment summary report**


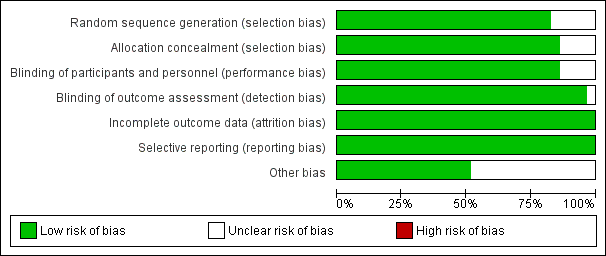


**S1/B Bias assessment graph**


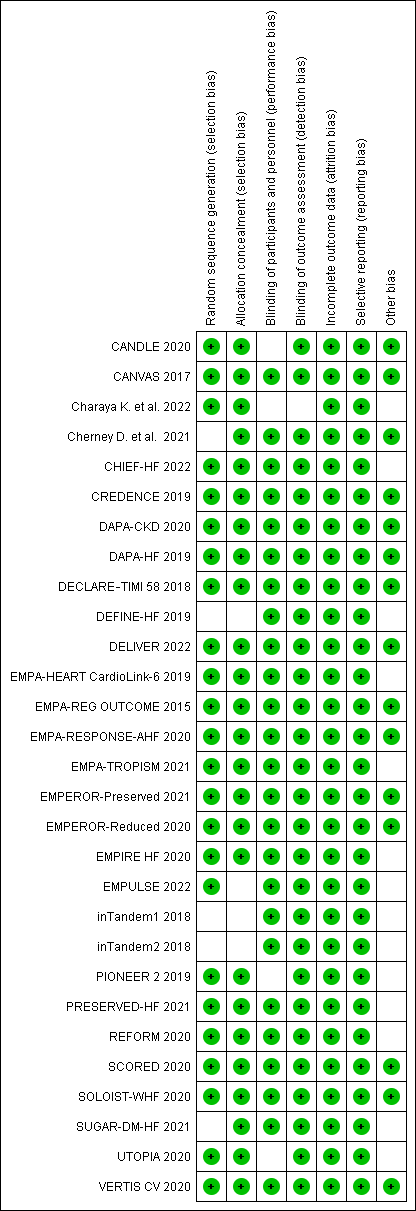


# **Figure S2. Visual estimation of publication bias with funnel plots**

The comparison-adjusted funnel plot showed no signs of important publication bias.

**Panel A: hospitalization for heart failure**

Egger’s regression test supports no publication bias (p = 0.1475)


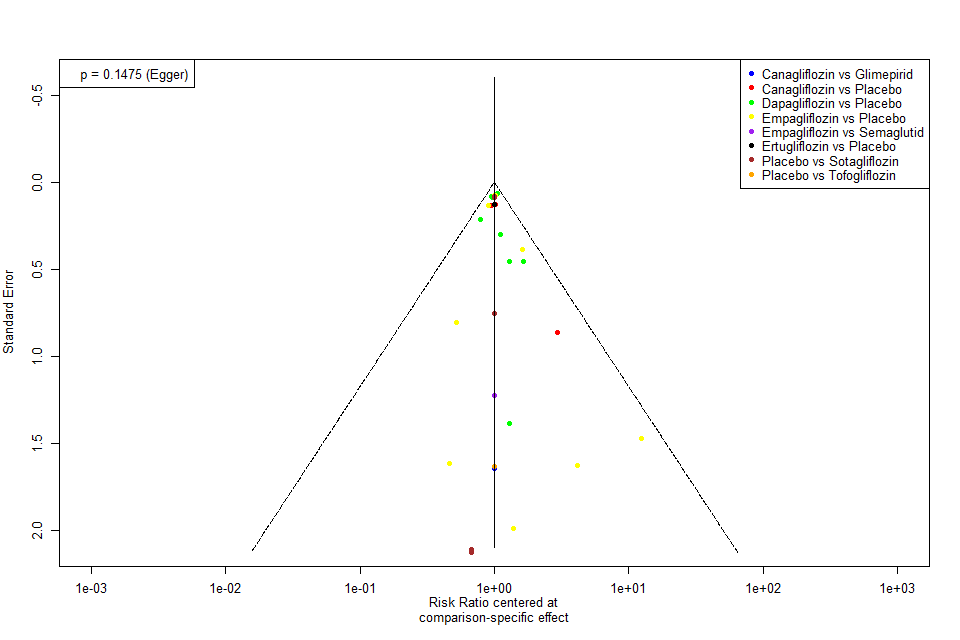


**Panel B: major adverse cardiac events**

Egger’s regression test supports no publication bias (p = 0.8877)


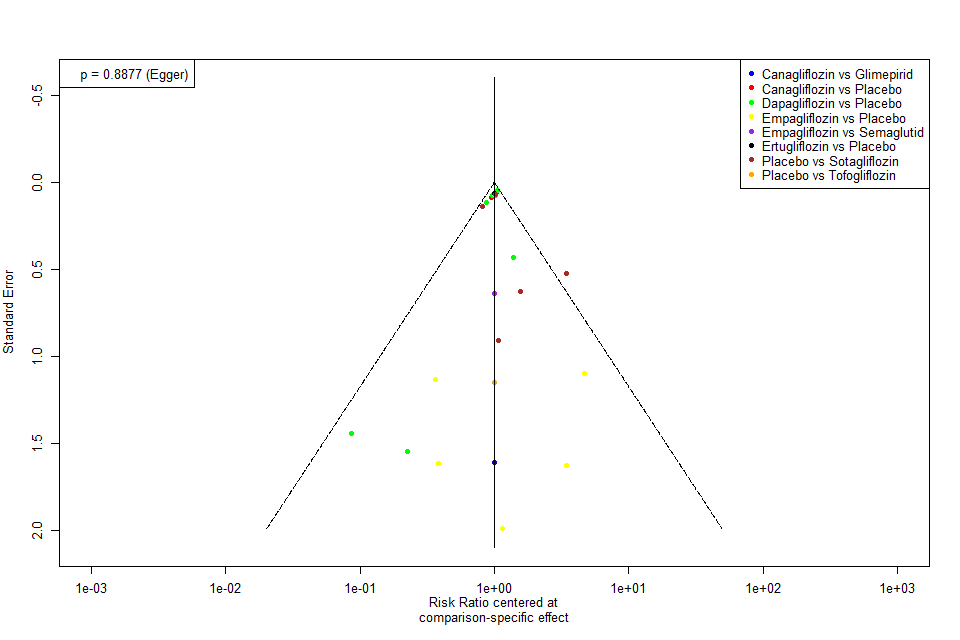


# **Figure S3. Summary of sex-specific subgroup analyses of the included trials**

Summary of sex-specific subgroup analyses from the included trials. The Forest plot shows the hazard ratio of the trials primary endpoint according to subgroups. Asterisk mark trials with more benefit with HR > 1.0.


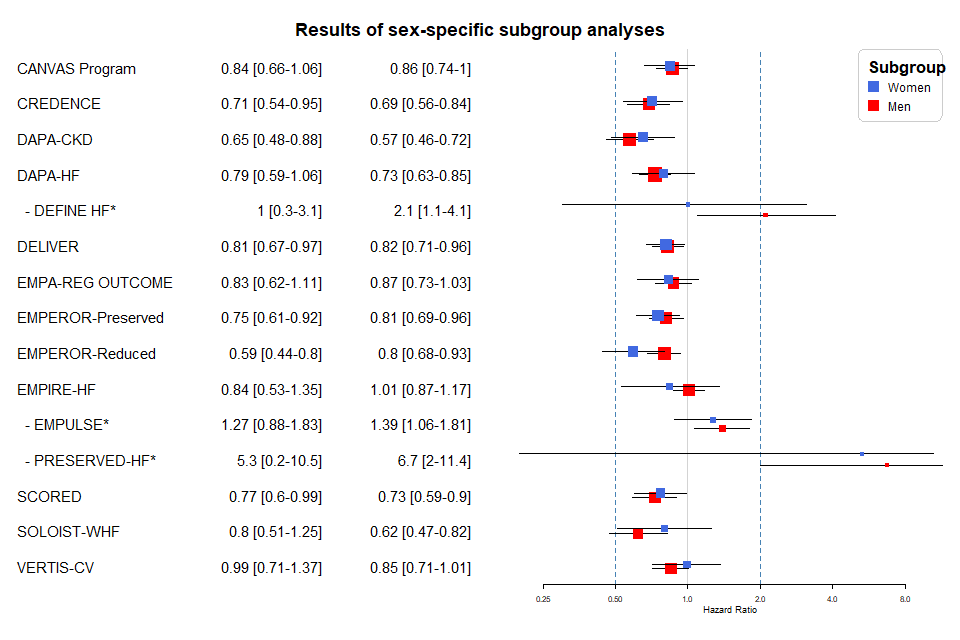


From the 29 included trials 16 comprised subgroup analyses, and 15 reported sex-specific data. It is important to note that these analyses reported only the relations related to the trial’s primary endpoint. (The only exception was EMPAREG OUTCOME where both primary outcome and CV mortality was analyzed)

From the 5 trials comparing dapagliflozin to placebo, 3 reported significant improvement with both sexes. (DELIVER, DAPA-CKD, PRESERVED-HF) In two the results of men were significantly better compared to the placebo, while with women this difference was not significant in one, and no difference was detected in another (DAPA-HF and DEFINE-HF, respectively). From the 5 trials of empagliflozin, in 2 trials both sexes had a significant benefit (EMPEROR-PRESERVED, EMPEROR-REDUCED), only men had a significant benefit in the EMPULSE trial. In the 2 other trials, the results were neutral or non-significantly better for both sexes. In canagliflozin and in sotagliflozin trials each had one trial with positive results for both sexes, and one showing significant benefit only in men (SCORED, SOLOIST-WHF, CREDENCE, CANVAS). VERTIS-CV compared ertugliflozin to placebo with a non-significant benefit in both sexes. Importantly, none of the trials reported an interaction that would support the existence of important sex-related interaction.

# **Citations of the included trials**

1. Zinman B, Wanner C, Lachin LM, Fitchett D, Bluhmki E, Hantel H, et al. Empagliflozin, Cardiovascular Outcomes, and Mortality in Type 2 Diabetes. N Engl J Med [Internet]. 2015 [cited 2022 Sep 9];373(22):17–8. Available from: https://pubmed.ncbi.nlm.nih.gov/26378978/

2. Wiviott SD, Raz I, Bonaca MP, Mosenzon O, Kato ET, Cahn A, et al. Dapagliflozin and Cardiovascular Outcomes in Type 2 Diabetes. N Engl J Med [Internet]. 2019 Jan 24 [cited 2022 Sep 9];380(4):347–57. Available from: https://pubmed.ncbi.nlm.nih.gov/30415602/

3. Voors AA, Angermann CE, Teerlink JR, Collins SP, Kosiborod M, Biegus J, et al. The SGLT2 inhibitor empagliflozin in patients hospitalized for acute heart failure: a multinational randomized trial. Nat Med [Internet]. 2022 Mar 1 [cited 2022 Sep 9];28(3):568–74. Available from: https://pubmed.ncbi.nlm.nih.gov/35228754/

4. Verma S, Mazer CD, Yan AT, Mason T, Garg V, Teoh H, et al. Effect of Empagliflozin on Left Ventricular Mass in Patients With Type 2 Diabetes Mellitus and Coronary Artery Disease: The EMPA-HEART CardioLink-6 Randomized Clinical Trial. Circulation [Internet]. 2019 Nov 19 [cited 2022 Sep 9];140(21):1693–702. Available from: https://pubmed.ncbi.nlm.nih.gov/31434508/

5. Tanaka A, Hisauchi I, Taguchi I, Sezai A, Toyoda S, Tomiyama H, et al. Effects of canagliflozin in patients with type 2 diabetes and chronic heart failure: a randomized trial (CANDLE). ESC Heart Fail [Internet]. 2020 Aug 1 [cited 2022 Sep 9];7(4):1585–94. Available from: https://pubmed.ncbi.nlm.nih.gov/32349193/

6. Spertus JA, Birmingham MC, Nassif M, Damaraju C v., Abbate A, Butler J, et al. The SGLT2 inhibitor canagliflozin in heart failure: the CHIEF-HF remote, patient-centered randomized trial. Nat Med [Internet]. 2022 Apr 1 [cited 2022 Sep 9];28(4):809–13. Available from: https://pubmed.ncbi.nlm.nih.gov/35228753/

7. Singh JSS, Mordi IR, Vickneson K, Fathi A, Donnan PT, Mohan M, et al. Dapagliflozin Versus Placebo on Left Ventricular Remodeling in Patients With Diabetes and Heart Failure: The REFORM Trial. Diabetes Care [Internet]. 2020 Jun 1 [cited 2022 Sep 9];43(6):1356–9. Available from: https://pubmed.ncbi.nlm.nih.gov/32245746/

8. Santos-Gallego CG, Vargas-Delgado AP, Requena-Ibanez JA, Garcia-Ropero A, Mancini D, Pinney S, et al. Randomized Trial of Empagliflozin in Nondiabetic Patients With Heart Failure and Reduced Ejection Fraction. J Am Coll Cardiol [Internet]. 2021 Jan 26 [cited 2022 Sep 9];77(3):243–55. Available from: https://pubmed.ncbi.nlm.nih.gov/33197559/

9. Rodbard HW, Rosenstock J, Canani LH, Deerochanawong C, Gumprecht J, Lindberg SØ, et al. Oral Semaglutide Versus Empagliflozin in Patients With Type 2 Diabetes Uncontrolled on Metformin: The PIONEER 2 Trial. Diabetes Care [Internet]. 2019 Dec 1 [cited 2022 Sep 9];42(12):2272–81. Available from: https://pubmed.ncbi.nlm.nih.gov/31530666/

10. Perkovic V, Jardine MJ, Neal B, Bompoint S, Heerspink HJL, Charytan DM, et al. Canagliflozin and Renal Outcomes in Type 2 Diabetes and Nephropathy. N Engl J Med [Internet]. 2019 Jun 13 [cited 2022 Sep 9];380(24):2295–306. Available from: https://pubmed.ncbi.nlm.nih.gov/30990260/

11. Neal B, Perkovic V, Mahaffey KW, de Zeeuw D, Fulcher G, Erondu N, et al. Canagliflozin and Cardiovascular and Renal Events in Type 2 Diabetes. N Engl J Med [Internet]. 2017 Aug 17 [cited 2022 Sep 9];377(7):644–57. Available from: https://pubmed.ncbi.nlm.nih.gov/28605608/

12. Nassif ME, Windsor SL, Borlaug BA, Kitzman DW, Shah SJ, Tang F, et al. The SGLT2 inhibitor dapagliflozin in heart failure with preserved ejection fraction: a multicenter randomized trial. Nat Med [Internet]. 2021 Nov 1 [cited 2022 Sep 9];27(11):1954–60. Available from: https://pubmed.ncbi.nlm.nih.gov/34711976/

13. Nassif ME, Windsor S, Tang F, Khariton Y, Husain M, Inzucchi S, et al. Dapagliflozin Effects on Biomarkers, Symptoms, and Functional Status in Patients With Heart Failure With Reduced Ejection Fraction: The DEFINE-HF Trial. Circulation [Internet]. 2019 Oct 1 [cited 2022 Sep 9];140(18). Available from: https://pubmed.ncbi.nlm.nih.gov/31524498/

14. McMurray JJV, Solomon SD, Inzucchi SE, Køber L, Kosiborod MN, Martinez FA, et al. Dapagliflozin in Patients with Heart Failure and Reduced Ejection Fraction. N Engl J Med [Internet]. 2019 Nov 21 [cited 2022 Sep 9];381(21):1995–2008. Available from: https://pubmed.ncbi.nlm.nih.gov/31535829/

15. Lee MMY, Brooksbank KJM, Wetherall K, Mangion K, Roditi G, Campbell RT, et al. Effect of Empagliflozin on Left Ventricular Volumes in Patients With Type 2 Diabetes, or Prediabetes, and Heart Failure With Reduced Ejection Fraction (SUGAR-DM-HF). Circulation [Internet]. 2021 Feb 9 [cited 2022 Sep 9];143(6):516–25. Available from: https://pubmed.ncbi.nlm.nih.gov/33186500/

16. Katakami N, Mita T, Yoshii H, Shiraiwa T, Yasuda T, Okada Y, et al. Tofogliflozin does not delay progression of carotid atherosclerosis in patients with type 2 diabetes: a prospective, randomized, open-label, parallel-group comparative study. Cardiovasc Diabetol [Internet]. 2020 Jul 9 [cited 2022 Sep 9];19(1). Available from: https://pubmed.ncbi.nlm.nih.gov/32646498/

17. Heerspink HJL, Stefánsson B v., Correa-Rotter R, Chertow GM, Greene T, Hou FF, et al. Dapagliflozin in Patients with Chronic Kidney Disease. N Engl J Med [Internet]. 2020 Oct 8 [cited 2022 Sep 9];383(15):1436–46. Available from: https://pubmed.ncbi.nlm.nih.gov/32970396/

18. Jensen J, Omar M, Kistorp C, Poulsen MK, Tuxen C, Gustafsson I, et al. Twelve weeks of treatment with empagliflozin in patients with heart failure and reduced ejection fraction: A double-blinded, randomized, and placebo-controlled trial. Am Heart J [Internet]. 2020 Oct 1 [cited 2022 Sep 9];228:47–56. Available from: https://pubmed.ncbi.nlm.nih.gov/32798787/

19. Packer M, Anker SD, Butler J, Filippatos G, Pocock SJ, Carson P, et al. Cardiovascular and Renal Outcomes with Empagliflozin in Heart Failure. N Engl J Med [Internet]. 2020 Oct 8 [cited 2022 Sep 9];383(15):1413–24. Available from: https://pubmed.ncbi.nlm.nih.gov/32865377/

20. Solomon SD, de Boer RA, DeMets D, Hernandez AF, Inzucchi SE, Kosiborod MN, et al. Dapagliflozin in heart failure with preserved and mildly reduced ejection fraction: rationale and design of the DELIVER trial. Eur J Heart Fail [Internet]. 2021 Jul 1 [cited 2022 Sep 9];23(7):1217–25. Available from: https://pubmed.ncbi.nlm.nih.gov/34051124/

21. Danne T, Cariou B, Banks P, Brandle M, Brath H, Franek E, et al. HbA 1c and Hypoglycemia Reductions at 24 and 52 Weeks With Sotagliflozin in Combination With Insulin in Adults With Type 1 Diabetes: The European inTandem2 Study. Diabetes Care [Internet]. 2018 Sep 1 [cited 2022 Sep 9];41(9):1981–90. Available from: https://pubmed.ncbi.nlm.nih.gov/29937431/

22. Damman K, Beusekamp JC, Boorsma EM, Swart HP, Smilde TDJ, Elvan A, et al. Randomized, double-blind, placebo-controlled, multicentre pilot study on the effects of empagliflozin on clinical outcomes in patients with acute decompensated heart failure (EMPA-RESPONSE-AHF). Eur J Heart Fail [Internet]. 2020 Apr 1 [cited 2022 Sep 9];22(4):713–22. Available from: https://pubmed.ncbi.nlm.nih.gov/31912605/

23. Cherney DZI, Ferrannini E, Umpierrez GE, Peters AL, Rosenstock J, Carroll AK, et al. Efficacy and safety of sotagliflozin in patients with type 2 diabetes and severe renal impairment. Diabetes Obes Metab [Internet]. 2021 Dec 1 [cited 2022 Sep 9];23(12):2632–42. Available from: https://pubmed.ncbi.nlm.nih.gov/34338408/

24. Charaya K, Shchekochikhin D, Andreev D, Dyachuk I, Tarasenko S, Poltavskaya M, et al. Impact of dapagliflozin treatment on renal function and diuretics use in acute heart failure: a pilot study. Open Heart [Internet]. 2022 May 24 [cited 2022 Sep 9];9(1). Available from: https://pubmed.ncbi.nlm.nih.gov/35609943/

25. Cannon CP, Pratley R, Dagogo-Jack S, Mancuso J, Huyck S, Masiukiewicz U, et al. Cardiovascular Outcomes with Ertugliflozin in Type 2 Diabetes. N Engl J Med [Internet]. 2020 Oct 8 [cited 2022 Sep 9];383(15):1425–35. Available from: https://pubmed.ncbi.nlm.nih.gov/32966714/

26. Buse JB, Garg SK, Rosenstock J, Bailey TS, Banks P, Bode BW, et al. Sotagliflozin in Combination With Optimized Insulin Therapy in Adults With Type 1 Diabetes: The North American inTandem1 Study. Diabetes Care [Internet]. 2018 Sep 1 [cited 2022 Sep 9];41(9):1970–80. Available from: https://pubmed.ncbi.nlm.nih.gov/29937430/

27. Bhatt DL, Szarek M, Steg PG, Cannon CP, Leiter LA, McGuire DK, et al. Sotagliflozin in Patients with Diabetes and Recent Worsening Heart Failure. N Engl J Med [Internet]. 2021 Jan 14 [cited 2022 Sep 9];384(2):117–28. Available from: https://pubmed.ncbi.nlm.nih.gov/33200892/

28. Bhatt DL, Szarek M, Pitt B, Cannon CP, Leiter LA, McGuire DK, et al. Sotagliflozin in Patients with Diabetes and Chronic Kidney Disease. N Engl J Med [Internet]. 2021 Jan 14 [cited 2022 Sep 9];384(2):129–39. Available from: https://pubmed.ncbi.nlm.nih.gov/33200891/

29. Anker SD, Butler J, Filippatos G, Ferreira JP, Bocchi E, Böhm M, et al. Empagliflozin in Heart Failure with a Preserved Ejection Fraction. N Engl J Med [Internet]. 2021 Oct 14 [cited 2022 Sep 9];385(16):1451–61. Available from: https://pubmed.ncbi.nlm.nih.gov/34449189/
